# Exploring Language Used in Posts on r/birthcontrol: Case Study Using Data From Reddit Posts and Natural Language Processing to Advance Contraception Research

**DOI:** 10.2196/46342

**Published:** 2023-06-30

**Authors:** Elizabeth Pleasants, Julia Holmes Ryan, Cheng Ren, Ndola Prata, Anu Manchikanti Gomez, Cassondra Marshall

**Affiliations:** 1 School of Public Health University of California Berkeley, CA United States; 2 School of Social Welfare University of California Berkeley, CA United States

**Keywords:** contraception, big data, Reddit, social networking site, contraceptive side effects, natural language processing, reproductive autonomy

## Abstract

**Background:**

Contraceptive choice is central to reproductive autonomy. The internet, including social networking sites like Reddit, is an important resource for people seeking contraceptive information and support. A subreddit dedicated to contraception, r/birthcontrol, provides a platform for people to post about contraception.

**Objective:**

This study explored the use of r/birthcontrol, from the inception of the subreddit through the end of 2020. We describe the web-based community, identify distinctive interests and themes based upon the textual content of posts, and explore the content of posts with the most user engagement (ie, “popular” posts).

**Methods:**

Data were obtained from the PushShift Reddit application programming interface from the establishment of r/birthcontrol to the start date of analysis (July 21, 2011, to December 31, 2020). User interactions within the subreddit were analyzed to describe community use over time, specifically the commonality of use based on the volume of posts, the length of posts (character count), and the proportion of posts with any and each flair applied. “Popular” posts on r/birthcontrol were determined based on the number of comments and “scores,” or upvotes minus downvotes; popular posts had 9 comments and a score of ≥3. Term Frequency-Inverse Document Frequency (TF-IDF) analyses were run on all posts with flairs applied, posts within each flair group, and popular posts within each flair group to characterize and compare the distinctive language used in each group.

**Results:**

There were 105,485 posts to r/birthcontrol during the study period, with the volume of posts increasing over time. Within the time frame for which flairs were available on r/birthcontrol (after February 4, 2016), users applied flairs to 78% (n=73,426) of posts. Most posts contained exclusively textual content (n=66,071, 96%), had comments (n=59,189, 86%), and had a score (n=66,071, 96%). Posts averaged 731 characters in length (median 555). “SideEffects!?” was the most frequently used flair overall (n=27,530, 40%), while “Experience” (n=719, 31%) and “SideEffects!?” (n=672, 29%) were most common among popular posts. TF-IDF analyses of all posts showed interest in contraceptive methods, menstrual experiences, timing, feelings, and unprotected sex. While TF-IDF results for posts with each flair varied, the contraceptive pill, menstrual experiences, and timing were discussed across flair groups. Among popular posts, intrauterine devices and contraceptive use experiences were often discussed.

**Conclusions:**

People commonly wrote about contraceptive side effects and experiences using methods, highlighting the value of r/birthcontrol as a space to post about aspects of contraceptive use that are not well addressed by clinical contraceptive counseling. The value of real-time, open-access data on contraceptive users’ interests is especially high given the shifting landscape of and increasing constraints on reproductive health care in the United States.

## Introduction

### Background

Contraceptive use is widespread in the United States, with approximately 65% of reproductive-age individuals (ages 15-49 years) using a form of contraception based on the 2017-2019 National Survey of Family Growth. Overall the use of contraception is relatively stable in the United States, with similar proportions of women using a method from 2008 to 2016 [1-3]. While the contraceptive pill has been and remains the most commonly used method of contraception in the United States (22% of all users in 2016), women’s use of long-acting reversible contraceptive methods such as contraceptive implants and intrauterine devices (IUDs) has increased since 2008, while the use of nonpill short-acting methods such as the contraceptive patch and ring has decreased [2,3].

Informed contraceptive choice, or the ability to freely access and choose from among the range of methods of fertility control, is recognized as a necessary component of reproductive autonomy, understood as people being able to control and decide if, when, and how to have children [4]. Contraceptive attributes—including efficacy for pregnancy prevention, duration, mode of delivery, and side effects—vary across methods. Users’ preferences for these attributes are central to the process of making informed contraceptive choices. For example, users often consider factors such as side effects of the method, perceptions of associated health risks, efficacy, and costs, along with personal values, relationship status, and desirability, and risk of pregnancy in the process of deciding about contraception [5-8]. Currently, best practices for contraceptive counseling involve emphasizing patient-centeredness and shared decision-making in response to an individual's needs, values, and preferences [9,10]. Despite these priorities, research shows that current training and recommendations for contraceptive counseling approaches can leave providers, staff, and potential users with significant knowledge gaps [9-14].

While patient-centered contraceptive counseling approaches can provide information to anticipate contraceptive use experiences, they have been found to have limited effects on method continuation [15]. Contraceptive users have unmet needs for responsive and experience-based information and support, including individual use experiences and management of method side effects [16,17]. Accordingly, potential contraceptive users often turn to sources beyond health care providers for contraceptive information, including peers and web-based resources. Peers are influential sources of contraceptive information [18,19], and individuals often consider contraceptive information received from peers as more trustworthy than clinicians [20-23]. The internet, which facilitates connection with geographically distant peers, has become an established source of health information, including healthy lifestyle advice and information related to treatment and diseases [24]. The number of individuals using the internet to search for health information continues to grow, a trend that has enabled people to engage more actively in their own health. Specific to contraception, research demonstrates that people are searching for information about a range of contraceptive methods, with variation across locations within the United States [25]. Research has noted the importance of web-based information about peers’ experiences for contraceptive decision-making [20,26-30].

Social networking sites, or web-based platforms designed to build social relationships, allow people to share experiences, ask questions, and seek support from others within a web-based community. Some scientists and experts argue that the anonymous nature of certain platforms makes them uniquely appealing to individuals who are seeking information about sensitive or stigmatized concerns like contraception [31-35] and may lead to the promotion of more authenticity and openness than other identifiable platforms or in-person interactions [36-38]. Web-based social communication may therefore be an important and rich source of data for studying contraceptive decision-making, needs, and preferences. Current research indicates that social networking sites are an important avenue for reproductive-age individuals to access information about sexual health, including contraceptive options. Studies have shown that people post on social networking sites about emergency contraception [39], withdrawal as a contraceptive method [31], novel methods of male contraception [40], prescription contraceptive methods generally [41], and experiences with IUDs specifically [42,43]. In addition to demonstrating that people use these platforms to discuss contraception, research also indicates that contraceptive information on social media can impact contraceptive preferences and use [18,44,45]. While this research demonstrates the importance of social networking sites to contraceptive experiences, there is a need for additional research leveraging data from these platforms, which may offer important insights into contraceptive use behaviors over time and concerns regarding contraceptive methods.

Reddit is a social media platform that houses web-based communities of anonymous users called “Redditors.” It is one of the most popular websites in the United States, largely because of its user-driven aggregation and community-based curation of web-based content [32,46]. Reddit combines web content, popular news, forum discussions, and a social network into 1 giant platform that is currently used by nearly a quarter of young US adults. Reddit operates as a pseudoanonymous platform where registered members can contribute to the site with content such as images, texts, videos, and links. As such, Reddit data can provide rich, user-created textual descriptions communicating users’ concerns, needs, and experiences. A subreddit is a microcommunity within Reddit, centered on a specific topic. The platform aims to create values as part of a community that exists in a subreddit. These values are communicated through the design of each subreddit, the rules of that subreddit, the approach taken by moderators for that community, and the flairs for that subreddit. Each subreddit is governed by a set of volunteer moderators or “mods” elected from within the community who set subreddit-specific guidelines for posting content, review posts to ensure that those guidelines are being met, and can delete noncompliant posts and ban deviant users. While there is limited research on how Reddit has been used related to contraception, previous research leveraged Reddit as a data source to understand the needs and experiences of contraceptive users during the COVID-19 pandemic. This study highlights that Reddit data offer a unique opportunity for researchers to understand the thoughts and experiences of reproductive-age individuals in near real time [47]. Further work is needed to more holistically understand how Reddit has been used in relation to contraception, looking at posts outside of the COVID-19 pandemic and leveraging machine learning methods that allow for analysis of more posts than thematic content analysis.

### Rationale and Specific Research Aims

This study focuses on the subreddit r/birthcontrol. In 2017, this subreddit consisted of 20,107 members, and 2.9% of its members identified as male, while 97.1% of them identified as female [48]. This study explored contraception discussions on r/birthcontrol, from the inception of the subreddit through the end of 2020, to gain a sense of how people engaged with and contributed to r/birthcontrol. This study aims to analyze posts shared in r/birthcontrol to (1) describe posts shared in this web-based community, (2) identify distinctive interests and themes based on the textual content of posts, and (3) identify and explore the textual content of posts with the most user engagement (ie, “popular” posts). Our analysis of voluntarily shared, unsolicited narratives from a large group of contraceptive users can offer real-time insights into contraceptive decision-making processes and experiences to provide information beyond what can be gleaned from data collected in clinical settings or through traditional research approaches.

## Methods

### Study Design

The use of machine learning, and natural language processing (NLP) specifically, presents an opportunity for contraception researchers to work efficiently with big data from social networking sites to generate knowledge about how people are using language across an entire body of text rather than a subset. Additionally, the use of NLP expands beyond traditional qualitative analyses of textual data from Reddit, like those presented by recent research on the use of social networking sites for reproductive health [40,49-51], to provide new perspectives on the ways technology has been and is currently being used.

### Data Extraction and Describing the Community

The subreddit r/birthcontrol was established on July 21, 2011, as a “place to discuss birth control methods.” This subreddit has 2 moderators who uphold the following moderators-created and enforced rules for contributing to the community: (1) not doctors, (2) be welcoming, (3) tact, (4) respect, (5) Reddiquette, (6) no selling, advertising, or surveys, and (7) factual comments. We extracted data from r/birthcontrol for this analysis using the PushShift Reddit API (application programming interface). PushShift is a data platform for social networking sites that started to collect Reddit data in 2015 [52]. This study used the PushShift API through the *PushShift.io API Wrapper* package in Python (version 3). The PushShift Reddit API team noted that PushShift has a greater size limit for downloaded content and better facilitates researchers’ use of Reddit data than the official Reddit API [52].

Data were pulled from July 21, 2011, the establishment of r/birthcontrol, to December 31, 2020, when this analysis began. On Reddit, “posts” are descriptive information generated and shared by a community member with no word limit and the option to share images, links, videos, and text. For each post, there is an option to apply 1 of any of the available pre-established “flairs” for that subreddit to each post. On Reddit, a user can apply a flair or a “tag” to their post before sharing within a subreddit. Users can choose the flair they think most accurately describes their post, designating the informational “group” for that post. Flairs are determined and set by subreddit moderators and operate as content “tags” for Reddit posts. Flairs for r/birthcontrol include “Experience,” “SideEffects!?,” “WhichMethod?,” “MistakeorRisk?,” “Howto?,” and “Educational.” Users can either select a single predefined flair per post or not apply a flair.

“Comments” are any response to a post from a community member. Community members also have the option to “upvote” or “downvote” posts and comments, indicating approval or disapproval of that content and contributing to the ranking of that content on the subreddit’s home page, with posts having more upvotes being raised closer to the top where other community members are more likely to view it. Comments and likes are 2 strong indicators showing user interactions on social media platforms. Usually, the more comments and likes, the more popular the post is. In the PushShift API, a “score” is presented as a proxy for the popularity of each post in this community, which is calculated as upvotes minus downvotes. User interactions within the subreddit—including the number of characters per post and comment, the contents of posts and comments, and the upvotes, downvotes, and “scores” of the Reddit posts—are compiled in the API.

Before exploring the textual data, we did some necessary preprocessing of the text, including cleaning stop words and lemmatization using the Natural Language Toolkit package (eg, went to go and children to child [53]). We also excluded posts that did not contain any text in the body of the post, were deleted (by the poster after submission), and were removed (by Reddit or subreddit moderators).

### Distinctive Interests and Themes

To describe the use of r/birthcontrol over time, we counted the number of posts by month, calculated the average length of the text per post, and determined the log of text length through the *Pandas* package. Then, we narrowed the data to only the time period for which flairs were active on r/birthcontrol (from February 4, 2016). Flairs were used as a foundation for coding categories of posts. First, duplicative flairs were consolidated, for example, some flairs share the same meaning but are slightly different in their text, as with missing punctuation. After this process, we were left with the previously described 5 main flairs on r/birthcontrol. After excluding posts with flairs that did not have text in the body of the post, we also calculated the volume of posts to which flairs were applied and the proportion of posts with *each flair* over time to explore how users were indexing their own posts, which can be understood as indicative of posters’ interest in communicating about common themes within this community. From the number of posts tagged with each flair by year, a proportional bar chart was generated to present the frequency with which each flair was applied to posts across years (2016-2020).

To show the most distinctive words and phrases (2-word phrases) in all textual content of posts to r/birthcontrol, we used Term Frequency-Inverse Document Frequency (TF-IDF). TF is term frequency, meaning the number of times the term appears in a document, similar to the bag-of-words approach, and it allowed us to determine how frequently words were used in the text of posts. IDF is the inverse document frequency, weighing words by how prevalent or rare they are in the entire corpus [54]. This approach decreases the weight of some high-frequency words in posts (eg, day and take), providing a list of “distinctive” words and phrases used across the corpus of posts. We used the TfidfVectorizer function from Python’s *sklearn* package to convert the corpus text (natural language) into vectors, a matrix of TF-IDF features (SciKitLearn). Within the TfidfVectorizer function, there is a parameter called ngram_range, which decides n-grams to be extracted. For example, for ngram_range of (1,1), it is unigrams, and ngram_range of (1,2) indicates unigrams and bigrams. In our setting, we used both (1,1) and (2,2).

Using findings from the TF-IDF analysis, we investigated the most distinctive words and phrases (“terms”) in a sample of r/birthcontrol posts with flairs applied (starting in 2016 with the introduction of flairs) and compiled a ranking of the top 10 unigrams and bigrams. Finally, the same process for determining top-ranked unigrams and bigrams was replicated for posts within each flair group since the introduction of flairs.

### Content of “Popular” Posts

To examine the posts with the greatest or most user engagement in the subreddit, we explored the number of comments and “scores,” calculated by subtracting the down-vote counts from the up-vote counts. We were interested in focusing analyses on approximately 10% of posts that constituted the most popular content on this subreddit. After exploring the number of comments and the “scores” of posts (upvote count minus downvote count), we decided to select posts with more than 9 comments and a score of 3 or higher, categorized as “popular posts” for this analysis [55]. Among “popular posts” that had text in the body of the post, the textual content of the posts was explored using TF-IDF. We then explored differences in content between the subset of popular posts and the full sample of posts with flairs applied using the same TF-IDF approach described above. Using the TF-IDF scores for popular posts in each flair group, a ranking of unigrams and bigrams in each flair group was created.

### Ethics Approval

As this research was conducted using public internet posting, rather than data from private forums or conversations, and no other nonpublic information was used, this study team operated following previously described approaches where ethics approvals were not deemed necessary [56].

## Results

### Web-Based Community and Key Terms

**Figure 1:**
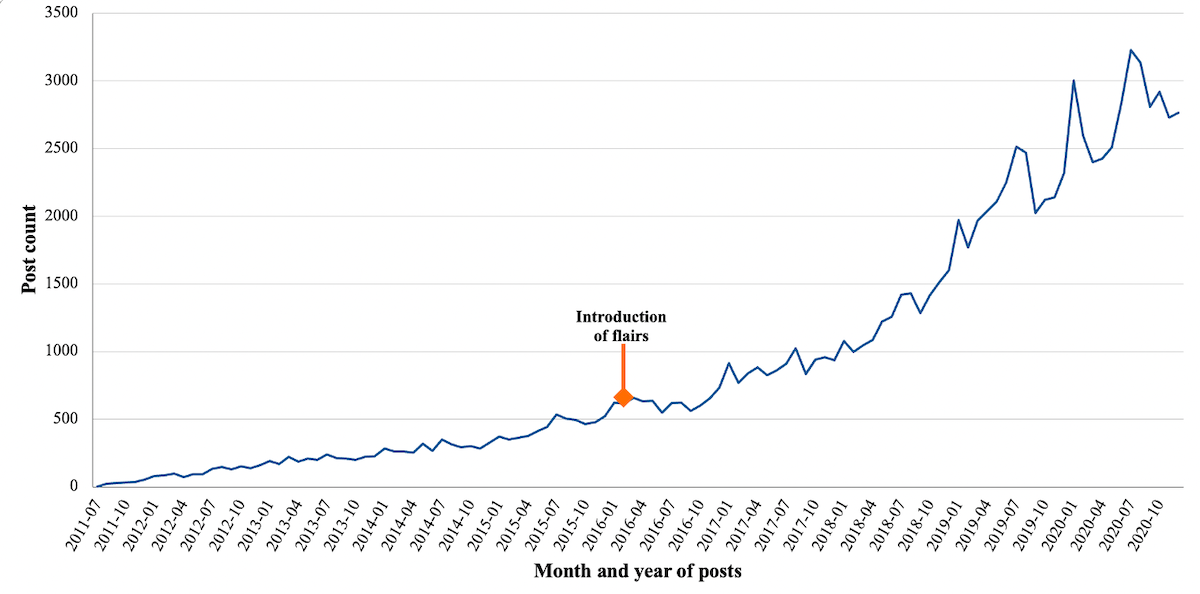
Volume of all monthly posts to r/birthcontrol over time (July 1, 2011, to December 31, 2020; total posts N=105,485).

Since its establishment in 2011, there were 105,485 total posts to r/birthcontrol. The number of monthly posts largely increased over time (Figure 1), with almost 3500 posts in July 2020.

Within the timeframe for which flairs were available on r/birthcontrol (after February 4, 2016), users applied flairs in this analysis to 78% (n=73,426) of posts. Applying exclusions to these posts, 1619 did not contain any text, 2746 were deleted by users, and 267 were removed. This left us with an analytic sample of 68,824 posts. The majority of posts were exclusively textual content (n=66,071, 96%), with only 4% (n=2753) including images. Posts averaged 731 characters in length, with a median of 555 characters. Overall, 86% (n=59,189) of posts had comments, with a median of 3 comments, and only 10% (n=6882) of posts with 10 or more comments. In total, 96% (n=66,071) of posts had a score, with only 2% (n=1376) having a score of more than 10.

### Distinctive Interests and Themes Within All Posts

**Figure 2:**
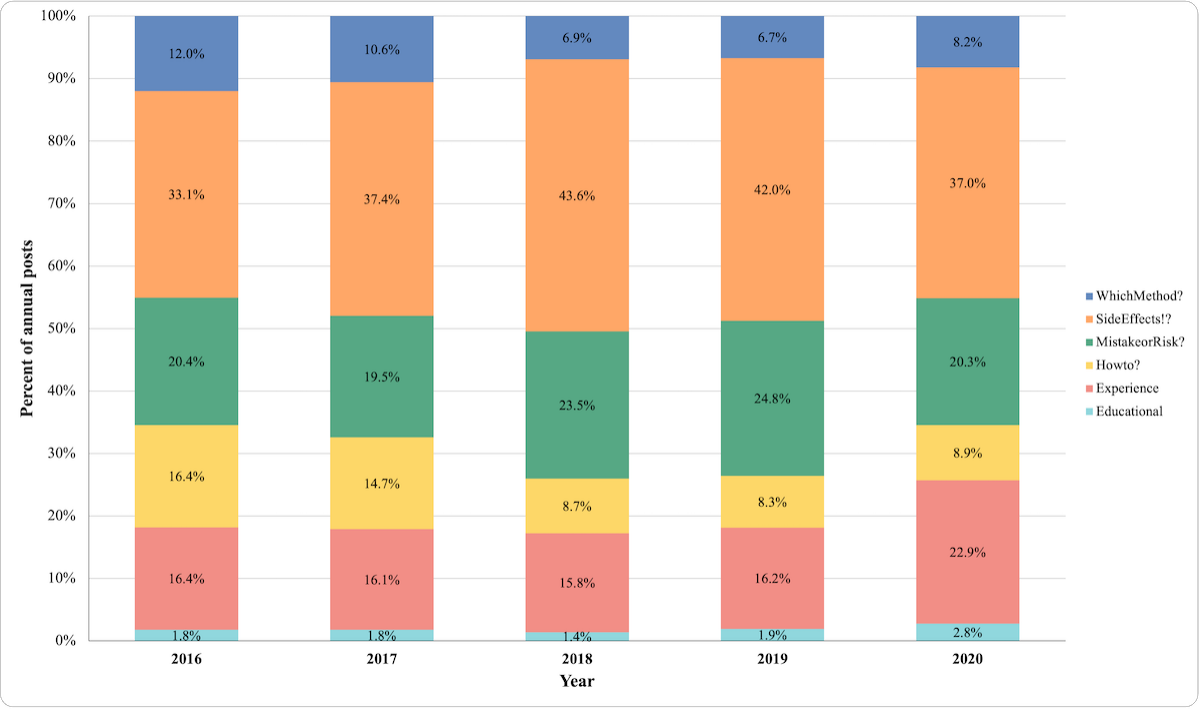
Proportion of r/birthcontrol posts with each flair applied by year among posts with flairs (2016-2020; total posts N=68,824).

The absolute number of posts to which each flair was applied increased over time, paralleling the increasing number of posts in this community between 2016 and 2020. The proportion of posts from each year to which each flair was applied is presented in Figure 2. Across all years, “SideEffects!?” was the most frequently used flair, applied to 40% (n=27,530) of posts, compared to 22% (n=15,141) for “MistakeorRisk?,” 19% (n=13,077) for “Experience,” 10% (n=6824) for “Howto?”, and 8% (n=5781) for “WhichMethod?”

The top 10 unigrams and bigrams among all r/birthcontrol posts with flairs (n=69,061) are presented in Table 1. The distinctive words (unigrams) were “pill,” followed by “period,” “day,” “take,” “week,” “get,” “month,” “start,” “IUD,” and “bleed.” The most distinctive 2-word phrases (bigrams) were “birth control,” followed by “take pill,” “feel like,” “copper IUD,” “control pill,” “week ago,” “unprotected sex,” “get period,” “day period,” and “month ago.” These top n-grams indicate that contraceptive methods, menstrual experiences, timing, feelings, and unprotected sex were the most distinctive themes described in posts in this community.

The top 10 unigrams and bigrams for posts with each flair applied are presented in Table 2. Across flair groups, “pill,” “period,” “day,” and “IUD” were consistently among the top 4 unigrams, with greater variation in distinctive words but at lower frequencies (ie, those with lower rankings). Similarly, “birth control” and “take pill” were top bigrams used across flair groups. But there was greater variation in top bigrams between groups, with specific concerns related to pregnancy risk seen among posts in “MistakeorRisk?” with bigrams “unprotected sex,” “miss pill,” “pregnancy test,” “take plan” (likely referring to taking Plan B emergency contraceptive), and “get pregnant.” Posts in “Howto?” seemed to largely consist of questions related to the use of oral contraceptive pills, with “start take,” “new pack,” “active pill,” “placebo pill,” “control pill,” and “placebo week” as top bigrams. Posts in “WhichMethod?” reflected user interest in the use of different methods with “copper IUD,” “hormonal IUD,” “take pill,” “control pill,” and “mini pill,” but also interest in the side effects and physical experiences of using different methods with “sex drive,” “weight gain,” “feel gain,” and “mood swing.”

**Table 1:** Top 10 weighted unigrams and bigrams for r/birthcontrol posts based on Term Frequency-Inverse Document Frequency analysis among posts with flairs applied (February 4, 2016 to December 31, 2020; total posts N=68,824).

Rank	Unigram	Bigram
1	Pill	Birth control
2	Period	Take pill
3	Day	Feel like
4	Take	Copper IUD^a^
5	Week	Control pill
6	Get	Week ago
7	Month	Unprotected sex
8	Start	Get period
9	IUD	Day period
10	Bleed	Month ago

^a^IUD: intrauterine device.

**Table 2:** Top 10 weighted unigrams and bigrams for r/birthcontrol posts by flair group based on Term Frequency-Inverse Document Frequency analysis (February 4, 2016, to December 31, 2020; N=68,824).

Rank	SideEffects!?			MistakeorRisk			Experience			Howto?			WhichMethod?	
	Unigram	Bigram	Unigram		Bigram	Unigram		Bigram	Unigram		Bigram	Unigram		Bigram
										
1	Period	Birth control	Pill		Birth control	Period		Birth control	Pill		Birth control	Pill		Birth control
2	Pill	Feel like	Day		Take pill	Pill		Copper IUD^a^	Period		Take pill	IUD		Copper IUD
3	Day	Take pill	Period		Unprotected sex	IUD		Feel like	Day		Start take	Period		Sex drive
4	Month	Week ago	Take		Miss pill	Get		Get IUD	Start		New pack	Control		Weight gain
5	Get	Month ago	Week		Pregnancy test	Day		Take pill	Take		Active pill	Birth		Feel like
6	Week	Copper IUD	Sex		New pack	Experience		Control pill	Week		Start new	Want		Take pill
7	Bleed	Get period	Time		Take plan	Month		Get period	Pack		Day period	Get		Control pill
8	Start	Day ago	Start		Pill day	Like		Sex drive	Month		Placebo pill	Year		Mood swing
9	Take	Start take	Get		Get pregnant	Cramp		Month ago	Time		Control pill	Like		Mini pill
10	IUD	Control pill	Month		Control pill	Take		Week ago	Control		Placebo week	Month		Hormonal IUD

^a^IUD: intrauterine device.

### Distinctive Interests and Themes Within “Popular” Posts

There were 2318 popular posts, for this research defined as posts with 9 or more comments and a score of 3 or more (Methods section), on r/birthcontrol posts since the introduction of flairs. “Experience” was the most commonly applied flair among top posts (n=719, 31%), followed by “SideEffects!?” (n=672, 29%), “WhichMethod?” (n=371, 16%), “MistakeorRisk?” (n=371, 16%), and “Howto?” (n=185, 8%). Compared to all posts, “Experience” and “WhichMethod?” were more common among popular posts.

The top 10 unigrams and bigrams for popular posts with each flair applied are presented in Table 3. While largely similar to the top n-grams seen across all posts, popular posts more often discussed IUDs in almost all flair groups (excluding “MistakeorRisk?”). Across flair groups, popular posts most often included “IUD,” “period,” and “pill” (similar to all posts). Within flair groups, n-grams for popular posts were as follows.

- For “SideEffects!?,” top n-grams were also commonly related to IUDs (“IUD,” “copper IUD,” and “get IUD”), birth control pills (“pill” and “control pill”), specific side effects (“sex drive,” “weight gain,” and “mood swing”), and timing (“month,” “day,” and “month ago”).
- For “MistakeorRisk?,” popular posts often discussed contraceptive methods (“pill,” “condom,” “IUD,” “take pill,” “copper IUD,” “use condom,” and “control pill”), unprotected sex and pregnancy risk (“get pregnant,” “unprotected sex,” and “pregnancy test”), and timing (“day,” “time,” and “week ago”).
- Among popular “Experience” posts, physical experience phrases were common (“cramp,” “feel,” “pain,” “feel like,” “felt like,” and “period cramp”), as were discussions of IUDs (“get IUD” and “copper IUD”). “Horror story,” “sex drive,” and “get pregnant” were the other top bigrams for “Experience” posts.
- For “Howto?,” popular posts were similar to those for “MistakeorRisk” but with more common discussion of IUDs and action or experience (“get,” “want,” “like,” “get IUD,” “feel like,” “planned parenthood,” “take pill,” and “IUD insert”).
- For popular posts in “WhichMethod?,” similar to all posts different methods were discussed (“IUD,” “pill,” “copper IUD,” “use condom,” “control pill,” and “hormonal control”), along with efficacy, side effects, and risk words (“sex drive,” “get pregnant,” “weight gain,” and “long term”).

**Table 3:** Top 10 weighted unigrams and bigrams for r/birthcontrol popular posts by flair group based on Term Frequency-Inverse Document Frequency analysis (February 4, 2016, to December 31, 2020; N=2318).

Rank	SideEffects!?			MistakeorRisk?			Experience			Howto?			WhichMethod?	
	Unigram	Bigram	Unigram		Bigram	Unigram		Bigram	Unigram		Bigram	Unigram		Bigram
										
1	Period	Birth control	Pill		Birth control	IUD^a^		Birth control	Pill		Birth control	IUD		Birth control
2	Pill	Copper IUD	Period		Get pregnant	Period		Copper IUD	Period		Copper IUD	Pill		Copper IUD
3	IUD	Feel like	Day		Take pill	Get		Feel like	IUD		Use condom	Control		Sex drive
4	Get	Sex drive	Take		Unprotected sex	Like		Get IUD	Get		Get IUD	Period		Feel like
5	Month	Weight gain	Condom		Pregnancy test	Experience		Sex drive	Day		Feel like	Birth		Get pregnant
6	Day	Mood swing	Get		Copper IUD	Cramp		Horror story	Know		Plan parenthood	Want		Weight gain
7	Like	Take pill	Sex		Use condom	Pill		Felt like	Week		Take pill	Get		Use condom
8	Experience	Month ago	IUD		Control pill	Day		Period cramp	Want		Control pill	Like		Long term
9	Control	Get IUD	Pregnant		Have sex	Feel		Take pill	Condom		Week ago	Year		Control pill
10	Feel	Control pill	Time		Week ago	Pain		Get pregnant	Like		IUD insert	Sex		Hormonal birth

^a^IUD: intrauterine device.

## Discussion

### Principal Results

This descriptive analysis of posts on the r/birthcontrol subreddit revealed several findings of interest. First, posts on r/birthcontrol have increased over time since the establishment of the subreddit in 2011, with a notable increase in the volume of posts in the last 5 years. Our findings show that r/birthcontrol was used to seek information about contraception, which aligns with past research demonstrating that the internet is a source of contraceptive information [25,28-30,39,41,42], and Reddit has been used to discuss sensitive health-related topics such as contraception, eating disorders, domestic abuse, HPV vaccination, and prenatal testing [32-35,47]. Engagement in web-based communities has been found to positively impact emotional and physical health outcomes in past research with new parents, and people with cancer, HIV, and mental health concerns [57-62]. Access to support is central to informed contraceptive decision-making and effective method use [27], but current clinical resources, including counseling approaches, leave gaps in support for many people considering or currently using contraception, particularly related to experiences of side effects [16,23,63]. r/birthcontrol exemplifies an established digital space where people seek to meet these support needs within a community of peers.

Second, leveraging the opportunity presented by user-applied flairs as a tool for categorizing the topical content of Reddit posts, we found that the use of flairs on r/birthcontrol has increased over time in parallel to the progressively increasing volume of posts. Of the 6 flairs explored, “SideEffects!?” was most frequently applied to posts across years, while among popular posts “Experience” and “SideEffects!?” were the most frequently applied flairs. This suggests that a high proportion of members of this subreddit were engaging with it to discuss side effects and contraceptive experiences, as they self-selected to apply these flairs to their posts. Knowledge about potential side effects is a key consideration for users who seek contraceptive information [5-8,27], and side effects can be a major deterrent to consistent and sustained contraceptive use [26,64,65]. While the experiences and priorities of prospective contraceptive users should be central to the decision-making process, that is not always the case with existing clinical counseling approaches that do not effectively address questions and concerns about side effects [9-12,14,16,66,67]. Our findings highlight that there are information and support needs related to contraceptive side effects that are not being met in clinical contexts.

Third, findings from the TF-IDF analyses shed additional light on distinctive interests posted in this community, including contraceptive methods, menstrual experiences, timing, feelings, and unprotected sex. While n-gram results for posts with each flair varied somewhat, topics including the contraceptive pill, menstrual experiences, and timing were discussed across flair groups. Based on TF-IDF results, IUDs and language related to experience using contraception (feelings, side effects, and method use) were more often discussed among popular posts than the entire sample of posts. Popular posts are presented at the top of the subreddit, and therefore, most seen by people engaging with this community—meaning that this content has the greatest potential to impact both community members and people “lurking” (or reading without actively engaging) on a subreddit. While IUD use has been increasing steadily over the past 15 years (with use among contraceptive users increasing from less than 3% in 2002 to 14% in 2018) [2,3,17] and IUDs are known to be safe and highly effective contraceptive methods [68], research shows that misconceptions and myths about IUDs persist—particularly among young and nulliparous people [69-71]. Our findings suggest that descriptive narratives, particularly those related to IUDs, are the most interacted with and have the greatest visibility on r/birthcontrol.

### Strengths, Limitations, and Future Directions

Reddit and data from health topic-specific subreddits not only serve as resources for individuals navigating health experiences and concerns but also present novel opportunities for researchers. This analysis of data from r/birthcontrol serves as a case study of how public health researchers can use NLP to explore the use of Reddit to communicate about health needs. NLP presents powerful tools for efficient and reproducible analysis of textual data, which allowed us to explore a large corpus of text across the history of a subreddit, but NLP does not provide the nuanced and contextualized insights gained from qualitative analytic approaches [72] and can introduce biases through the analytic approaches used and the decisions made throughout the research process [73-75]. The use of TF-IDF as an exploratory and descriptive tool for this analysis provides important insights into the structures of communication in this community and how that changed over time. Future research should build upon this work to expand our descriptive findings, integrating traditional qualitative analytic approaches with NLP, as with the use of NLP to support partial automation of qualitative coding [76]. Provided that researchers are aware of the foundations and limitations of various NLP tools, using methods beyond TF-IDF along with qualitative coding could facilitate exploring not only the language being used across the body of posts but also the contextual and descriptive information shared over time.

Additionally, while using user-applied flairs as a categorization tool allowed us to explore broader interests across posts and differences in post content between flair groups, the available flairs for each subreddit are determined by moderators, and the application of flairs is highly subjective. As such, these findings are limited by the flairs made available in r/birthcontrol and the level of consistency with which flairs were applied by posters. Future research could explore how flairs are applied more deeply to provide additional insights into their power as a categorization tool. Despite this limitation, our research approach provides an accessible method to categorize content shared on Reddit.

It is important to understand not just how web-based communities are used by posters, but how they impact community members and their health. While our analysis focused exclusively on posts to r/birthcontrol, analysis of comments shared in this community could provide additional insights into the impacts of this community on contraceptive outcomes as well as the dynamics of communication within the community. Furthermore, estimates indicate that as much as 98% of Reddit users only observe and never post or comment (“lurkers” [77]), meaning that analyzing posts to this community can provide insights into the content seen by many users but with limited information about their concerns and needs. By focusing part of our analysis on popular posts, we sought to explore the content most likely to be seen by all users, but further research should consider looking beyond the content posted on Reddit to explore how engaging with this community might shape contraceptive knowledge, attitudes, and behaviors for contributors and lurkers. Additionally, this research looked at posts since the establishment of flairs on r/birthcontrol in 2016 as a cohesive time frame for our sample. Given the power of Reddit data as a resource for tracking trends over time, future research should also explore how people’s use of this type of community changed over time.

As a pseudo-anonymous platform where users are not required to create profiles and are only identified by usernames, Reddit provides a space where users feel comfortable discussing sensitive and stigmatized topics [32-36,40]. While this means that r/birthcontrol likely operates as an appealing space for people to seek and share support related to contraception, it also means that we are unable to explore the demographics of members of the r/birthcontrol community and therefore any relationships between experiences of oppression related to identity and how individuals used this community. Overall, Reddit is a primarily young, White, educated, and male social networking site [78], which presents concerns about the representativeness and generalizability of our findings for other contraceptive users. In contrast to the demographics of the overall Reddit user population, we do know that this community is primarily women based on recent estimates [48] and believe that it may differ in other important ways from users of other subreddits given the specific focus of r/birthcontrol. While we cannot say that the interests and needs of people posting on r/birthcontrol represent those of the general population, this community presents the opportunity for engagement from geographically dispersed individuals with varied backgrounds and contraceptive experiences. As such, our findings provide insights into important considerations for the provision of contraceptive care in a world where the internet is a part of people’s information-seeking and decision-making processes.

Finally, while this research established the range of contraceptive interests and concerns across posts to r/birthcontrol, it did not explore the presence and content of misconceptions and misinformation in the community. Future research on the use of r/birthcontrol and social networking sites for contraception more generally should explore what misconceptions are commonly described and how they diffuse within communities. For prospective users, misinformation and misconceptions about contraceptive methods are known to be widespread, including those relating to the perceived safety and efficacy of certain methods [69-71,79]. While misconceptions about contraception are known to shape contraceptive decision-making and use [44,80,81], the impacts of web-based contraceptive misinformation on users are not well understood. Understanding misconceptions and uncertainty, particularly around the safety and efficacy of contraceptive methods, and the effects of misconceptions on contraceptive choices through content from social networking sites, could provide new avenues for understanding and addressing these misconceptions and facilitating informed contraceptive decision-making.

### Conclusions

This study presents an analysis of post data from r/birthcontrol, providing insights into how this digitally facilitated web-based community was used from 2011 to 2020. We found that people commonly wrote about contraceptive side effects and experiences using methods, highlighting the value of this subreddit as a space to post about aspects of contraceptive use that are not well addressed by clinical contraceptive counseling. The value of real-time, open-access data on contraceptive users' interests is especially high given the shifting landscape of and increasing constraints on reproductive health care in the United States, creating opportunities to understand population interests and current needs for contraceptive support.

## Abbreviations

API: application programming interface
IUD: intrauterine device
NLP: natural language processing
TF-IDF: Term Frequency-Inverse Document Frequency

## Data Availability

All data used for this analysis were obtained through a publicly available application programming interface (PushShift’s Reddit API) using Python. Data are accessible through the Harvard Dataverse in a public repository [82].
